# Comparison of Experimental Diabetic Periodontitis Induced by* Porphyromonas gingivalis* in Mice

**DOI:** 10.1155/2016/4840203

**Published:** 2016-11-23

**Authors:** Qi Wang, Peng Zhang, Ray Aprecio, Dongjiao Zhang, Hao Li, Ning Ji, Omaima Mohamed, Wu Zhang, Yiming Li, Yi Ding

**Affiliations:** ^1^State Key Laboratory of Oral Diseases, West China Hospital of Stomatology, Sichuan University, No. 14, 3rd Section S. Renmin Road, Chengdu, China; ^2^Center for Dental Research, School of Dentistry, Loma Linda University, 11175 Campus Street, Loma Linda, CA, USA; ^3^Shandong Provincial Key Laboratory of Oral Tissue Regeneration, School of Stomatology, Shandong University, 44-1 Wenhua W. Road, Jinan, China; ^4^Department of Prosthodontics, The Affiliated Hospital of Stomatology, Guangxi Medical University, 10 Shuangyong Road, Nanning 530021, China

## Abstract

Periodontitis is one of the severe complications in diabetic patients and gingival epithelium plays an initial role on the onset and progression of this disease. However the potential mechanism is yet sufficiently understood. Meanwhile, the research on the correlational experimental animal models was also insufficient. Here, we established periodontitis with type 2 diabetes in* db/db* and Tallyho/JngJ (TH) mice and periodontitis with type 1 diabetes in streptozotocin induced diabetes C57BL/6J (STZ-C57) mice by oral infection of periodontal pathogen* Porphyromonas gingivalis* W50. We demonstrated that periodontal infected mice with high blood glucose levels showed dramatically more alveolar bone loss than their counterparts, in which infected* db/db* mice exhibited the most bone defects. No contrary impact could be observed between this periodontal infection and onset and severity of diabetes. The expressions of PTPN2 were inhibited whereas the expression of JAK1, STAT1, and STAT3 increased dramatically in gingival epithelia and the serum TNF-*α* also significantly increased in the mice with diabetic periodontitis. Our results indicated that the variations of inflammation-related protein expressions in gingival epithelia might lead to the phenotype differences in the mice with diabetic periodontitis.

## 1. Introduction

Periodontitis, a worldwide complication of diabetes (known as diabetic periodontitis), is diagnosed by destruction of periodontal cementum, ligament, and alveolar bone [1]. Both types of diabetes in clinic, type 1 diabetes (T1D) and type 2 diabetes (T2D), exhibit dramatically higher risk and severity of periodontitis [2–4]. In recent years, researches are focused on the bidirectional communication between diabetes and periodontitis [5]. However, the underlying mechanism and the potential therapy are yet understood. Because of the complexity of clinical trials, establishing reliable animal models is particularly necessary.

In diabetic periodontitis, significantly increased inflammatory cytokines could be found in serum and gingival cervical fluid [6]. Previous studies have confirmed immune and inflammatory responses in the gingival epithelium play an initial role on the onset and development of diabetic periodontitis [7, 8]. Protein tyrosine phosphatase nonreceptor type 2 (PTPN2) is an intracellular tyrosine-specific phosphatase, which is expressed in epithelial cells, fibroblasts, or endothelial cells [9]. The biological function of PTPN2 is believed to vary in response to proinflammatory stimuli such as interferon-gamma (IFN-*γ*), tumor necrosis factor (TNF-*α*), hyperosmotic stress, or hyperglycemia [10, 11]. Moreover, proteins on Janus family kinase (JAK)/signal transducer and activator of transcription (STAT) pathway, an important signaling in inflammation, have recently been found to be targets of dephosphorylation by PTPN2 [12]. Inactivation of those substrates by dephosphorylation lead to the negative regulation of signaling pathways involved in inflammatory responses induced by the proinflammatory cytokines like TNF-*α*, which is also increased in serum and gingival cervical fluid of patients with periodontitis [13].

Mice are usually used as the experimental animal models for the research of human disease, because of its unique host response [14]. A large number of researchers have studied the experimental mice models of chronic periodontitis and diabetes disease, respectively [15, 16]. But the research about the establishment and characteristics of the experimental diabetic periodontitis mice models is yet insufficient. In this study, we established three mice models of experimental diabetic periodontitis and compared characteristics in metabolism and periodontal inflammation. The levels of PTPN2 expression and key factors on JAK/STAT pathway were analyzed to reveal the potential biological reasons behind the differences.

## 2. Materials and Methods

### 2.1. Experimental Animals

Twenty 4-week male* db/db* mice, 20 male Tallyho/JngJ (TH) mice, and 40 male C57BL/6J (C57) mice were fed commercial mouse food (finely ground autoclaved low-fat diet) and housed under a controlled environment (temperature around 22°C and relative humidity 45–55% with a 12-12-hour light-dark cycle) for the entire research process. At 6 week, 20 C57 mice were received streptozotocin (STZ, 55 mg/kg body weight; Sigma-Aldrich, St. Louis, MO, USA) for 5 days successively by intraperitoneal injection to establish diabetic mice (STZ-C57). Animal treatment was approved by the Ethics Committee of Animal Welfare (WCCSIRB-2015-133).

### 2.2. Study Design

All mice were randomly divided to infection and shame-infection group.* Porphyromonas gingivalis* (*P. gingivalis*) W50 was acquired from the State Key Laboratory of Oral Diseases, Sichuan University and cultured anaerobically in blood-agar (Oxoid, Oxoid Ltd., Hampshire, England) with hemin/menadione (Sigma-Aldrich).

At 6 weeks, the mice in the infection groups were inoculated with* P. gingivalis* as the following methods [17]: 10^9^ colony-forming units of* P. gingivalis* were diffused in 100 *μ*L phosphate buffered saline (PBS) with 2% carboxymethylcellulose and then orally inoculated three times every second day. The mice in shame-infection groups were infected with the equal volume of PBS (100 *μ*L). All mice were killed at 18 week.

### 2.3. Measurement of Fasting Blood Glucose and Body Weight Levels

Fasting blood glucose was determined every 2 weeks in tails blood collected from tail veins of the mice after an eight-hour fast using the glucose meter (OneTouch; LifeScan, Milpitas, CA, USA). Body weight was also determined every 2 weeks during the experimental process.

### 2.4. Determination of Serum Tumor Necrosis Factor-Alpha (TNF-*α*) Levels

At sacrifice, the serum tumor necrosis factor-alpha (TNF-*α*) levels were determined in tails blood collected as above in triplicate by ELISA kit (CUSABIO; Sino-American Biotechnology, Wuhan, China) according to manufacturer's instructions.

### 2.5. Quantification of Alveolar Bone Loss

Level of alveolar bone loss was determined using SEM (scanning electronic microscope; Zeiss EVO, CRAIC Technologies Inc., Kirchdorf, Germany) [18]. The alveolar bone loss level was measured by the average area (mm^2^) bordered as the cementoenamel junction, the mesial and distal line angles, and the alveolar bone crest on the lingual sides of the mandibular second molars [19]. Three evaluators calculated the data of same samples in a blinded, random fashion.

### 2.6. Immunohistochemical Analysis of Gingival Epithelium

Maxillas of* db/db* and C57 mice were dissected after sacrifice and were made into section slides and stained with immunohistochemical (IHC) as presented previously [20]. In short, both sides of maxillas were decalcified in 10% EDTA solution (BioRad, BioRad Laboratories, Hercules, CA) for 14 days and embedded in paraffin. The paraffin-embedded tissues were cut into thin sections (4 *μ*m) and stained with immunohistochemical. The primary antibodies, anti-PTPN2 (1 : 50), anti-JAK1 (1 : 100), anti-STAT1 (1 : 100), and anti-STAT3 (1 : 100), and the secondary antibodies (1 : 1000) were all from Santa Cruz Biotechnology (Santa Cruz, Santa Cruz Biotechnology, Inc., Santa Cruz, CA). Five slides were applied for each sample at 10 intervals. The staining pictures were captured by an optical microscope (Nikon 80i, Nikon Ltd., Tokyo, Japan).

### 2.7. Histological Examination of Pancreas Tissue

Pancreas tissues of four types mice were dissected, rinsed in PBS, and fixed in 4% paraformaldehyde (Sigma-Aldrich) for 24 hours. The paraffin-embedded tissue samples were cut into a series of 4 *μ*m section slides and stained with hematoxylin and eosin (Sigma-Aldrich). The morphological characteristics of pancreas tissues were observed in the images captured by an optical microscope (Nikon 80i).

### 2.8. Statistical Analysis

Data were shown as mean ± standard error of the mean (SEM) and analyzed with Student *t*-test when comparing two groups or one-way analysis of variance (ANOVA) test followed by SNK-*q* multiple comparisons when comparing three or more groups using SPSS software (SPSS 17.0, Chicago, IL, USA). A value of *P* < 0.05 was considered statistically significant.

## 3. Results

### 3.1. Different Metabolic Characters Were Shown in Four Types of Mice

**Figure 1:**
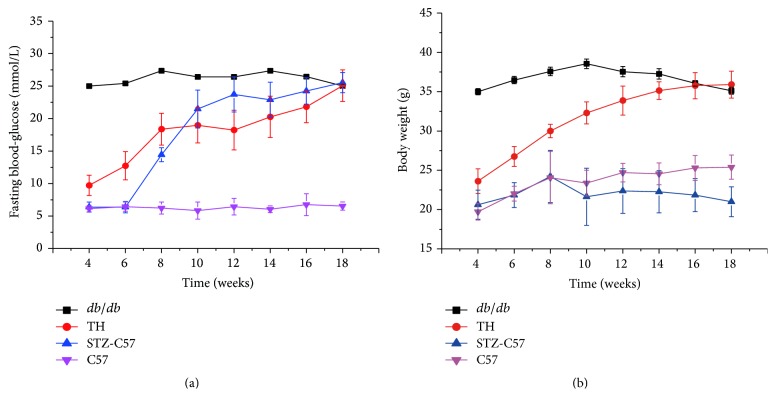
Fasting blood glucose (a) and body weight (b) levels in the infection groups of different mice. Values for each time point are expressed as mean ± SEM.

The fasting blood glucose and body weight levels of all mice were determined every 2 weeks and changes over time are presented (Figure 1). Within the mice with* P. gingivalis* infection,* db/db* mice exhibited hyperglycemia from the beginning of research and the fasting blood glucose was maintained at a high value as averagely 26 mmol/L during the experimental period. TH mice increased spontaneously and exhibited high glucose at 8 weeks old and then elevated continuously to a final value as approximately 25 mmol/L at sacrifice. STZ-C57 mice exhibited hyperglycemia 2 weeks after STZ injection at 8 weeks old and continued to increase from weeks 8–10. The level maintained steady as evenly 25 mmol/L from weeks 10–18, while the fasting blood glucose level of C57 mice maintained normal from 6 mmol/L to 7 mmol/L for the entire experimental period. The body weight level of* db/db* mice increased spontaneously from weeks 6–10 but decreased gradually after 10 weeks. Conversely, the body weight level of TH mice increased gradually during the research. The body weight level of C57 mice was steady, but the level of STZ-C57 mice in the infection groups decreased.

### 3.2. *P. gingivalis* Infection Did Not Affect the Fasting Blood Glucose Level among the Four Types of Mice

**Figure 2:**
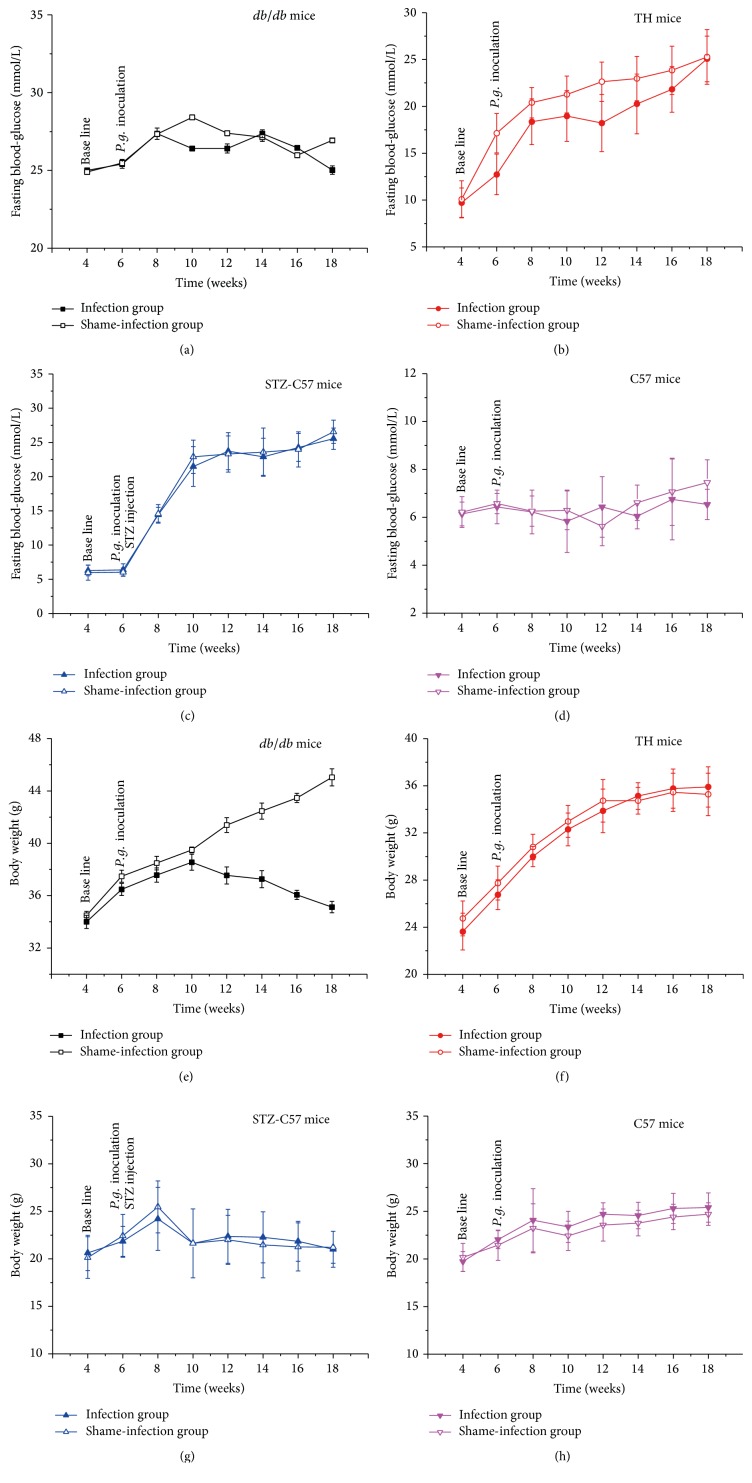
Comparison of fasting blood glucose and body weight levels in different mice: (a, e)* db/db* mice; (b, f) TH mice; (c, g) STZ-C57 mice; (d, h) C57 mice. No statistical difference (*P* > 0.05) detected for the fasting glucose levels between infection and shame-infection groups in four types of mice. At sacrifice the body weights of infected* db/db* were significantly higher than their controls (*P* < 0.01). No significant difference was found in the body weight between infection and shame-infection groups in TH, STZ-C57, and C57 mice. Values for each time point are expressed as mean ± SEM.

As shown in Figure 2, comparing infection groups with shame-infection groups, no statistical difference was observed in the fasting glucose levels among the four types of mice (*P* > 0.05), which indicated that* P. gingivalis* infection did not affect the metabolic feature in the mice. However, the body weight of the* db/db* infected mice exhibited a gradual decrease since weeks 10, while the shame-infection group continued to increase. At sacrifice the body weight of infection group was dramatically higher than that of shame-infection group in* db/db* mice (*P* < 0.01).

### 3.3. High Glucose Aggravated the Alveolar Bone Loss after* P. gingivalis* Infection among the Four Types of Mice

**Figure 3:**
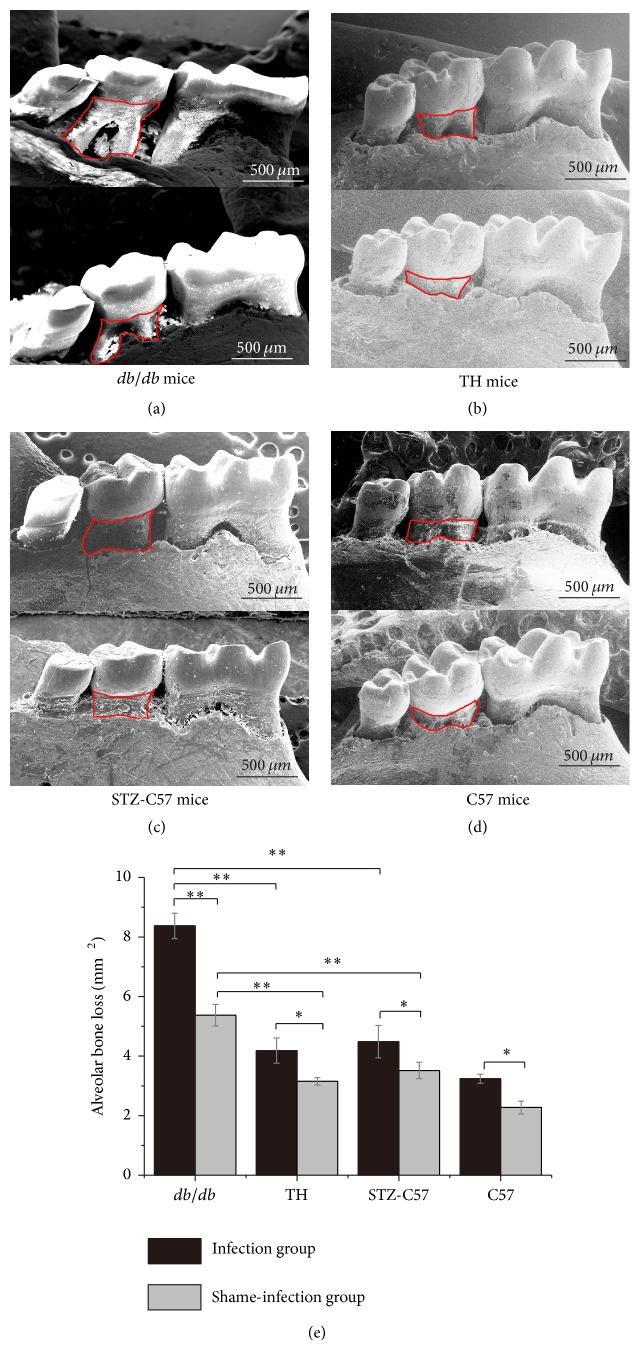
SEM images of mandibular jaws from mice in all groups: (a)* db/db* mice; (b) TH mice; (c) STZ-C57 mice; (d) C57 mice. Area within the red line was calculated as bone loss on the lingual sides of the mandibular second molars. (e) Comparison of alveolar bone loss level in different mouse models (^*∗*^ *P* < 0.05; ^*∗∗*^ *P* < 0.01).

The level of alveolar bone loss was significantly higher in injection groups compared to shame-injection groups (Figure 3). Within the infection groups,* db/db* mice exhibited more alveolar bone loss than those of TH mice, STZ-C57 mice, and C57 mice (*db/db* mice* versus* TH mice, STZ-C57 mice, and C57 mice: *P* < 0.01). Similar results could be found in the shame-infection groups (*db/db* mice* versus* TH mice, STZ-C57 mice, and C57 mice: *P* < 0.05).

### 3.4. Different Serum TNF-*α* Level Were Found among the Four Types of Mice

**Figure 4:**
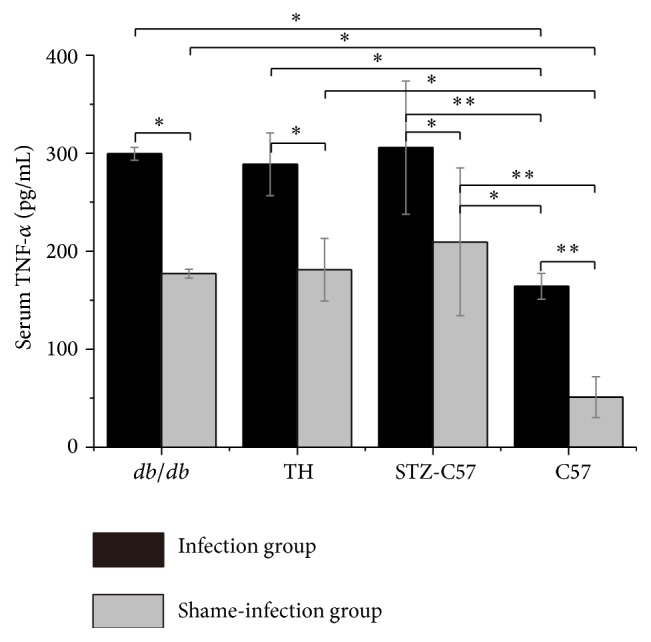
Serum TNF-*α* level at time of sacrifice determined by ELISA (^*∗*^ *P* < 0.05; ^*∗∗*^ *P* < 0.01).

As shown in Figure 4, the levels of serum TNF-*α* after sacrifice were higher in the infection groups compared to the shame-infection groups (C57 mice: *P* < 0.01; STZ-C57 mice: *P* < 0.05; TH mice: *P* < 0.05;* db/db* mice: *P* < 0.05). Within the infection groups, C57 mice exhibited less serum TNF-*α* levels than those of diabetic mice (STZ-C57, TH,* db/db* mice* versus* C57 mice: *P* < 0.01). Similar results could be observed in the shame-infected groups (STZ-C57, TH,* db/db* mice* versus* C57 mice: *P* < 0.01). No statistical difference was found in the serum TNF-*α* levels among diabetic mice in neither the infection nor the shame-infection groups (*P* > 0.05).

### 3.5. Alternations of Inflammation-Related Proteins Were Found in Gingival Epithelial Tissues

**Figure 5:**
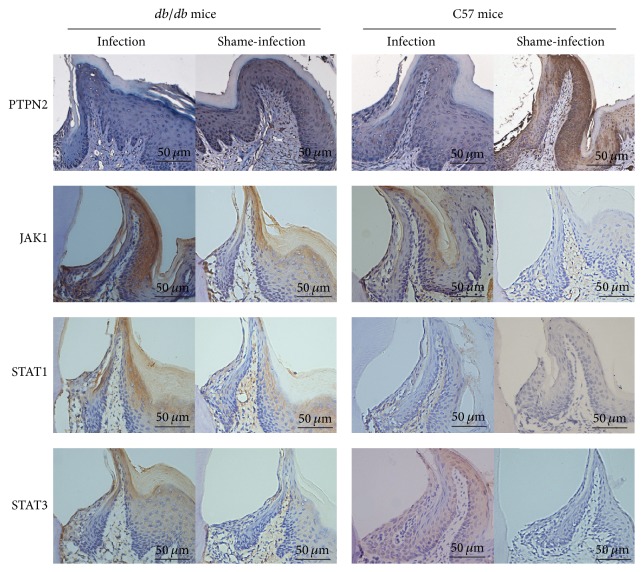
Protein expression of PTPN2, JAK1, STAT1, and STAT3 in the gingival epithelium of* db/db* and C57 mice was shown in the IHC staining images. Attenuated expression of PTPN2 and elevated expression of JAK1, STAT1, and STAT3 were seen in infection groups compared to shame-infection groups in both* db/db* and C57 mice.

To further illuminate the underlying mechanism of the alveolar bone loss in these mice models, we evaluated the protein expression of PTPN2, JAK1, STAT1, and STAT3 in gingival epithelium at sacrifice (Figure 5). C57 mice share the same genetic background with* db/db* mice and are often used as controls to study the diseases mechanism. The results demonstrated that high glucose seriously reduced PTPN2 expression in gingival epithelia of diabetic periodontitis mice. JAK1, STAT1, and STAT3 expression levels were dramatically elevated in infection groups of both* db/db* and C57 mice. Moreover, infected* db/db* mice exhibited the lowest PTPN2 expression compared to their shame-infection controls and C57 mice.

### 3.6. Pancreas Morphology of T1D and T2D Was Observed in the Four Types of Mice

**Figure 6:**
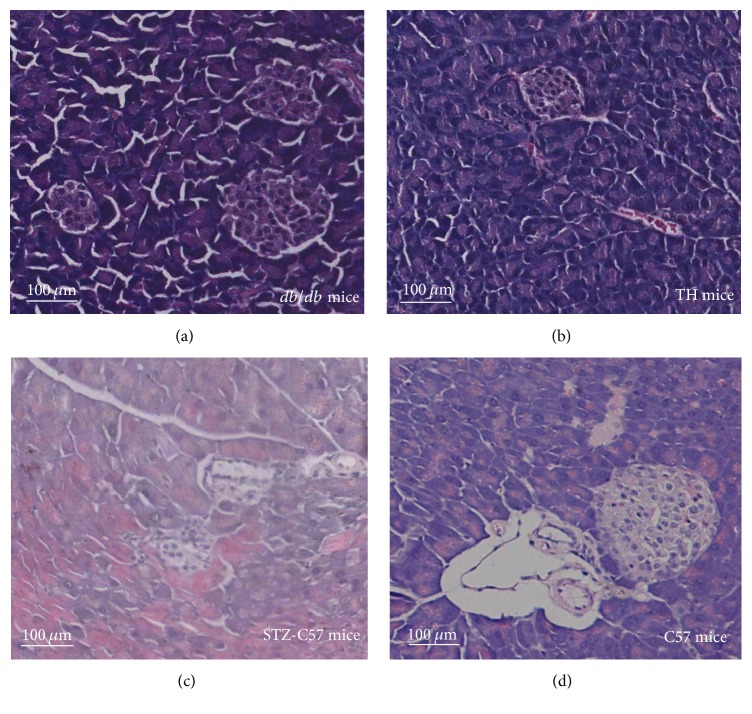
Pancreas morphology featured T1D and T2D in the four types of mice were observed in HE staining images.

As shown in Figure 6, in C57 mice, round or oval islets cells clusters with clear border were located in the central part of islets and contained a large number of islets *β* cells with round nuclei. In* db/db* mice and TH mice, the volume of islets cells clusters shrinked slightly; vessels enlarge mildly, which were the histological appearances of type 2 diabetes, while in STZ-C57 mice, the volume of islets clusters decreased dramatically. Vacuolar degeneration, necrosis, or disappearance in islets *β* cells and proliferous fibrotic tissue between islets *β* cells could be observed; thus the islets appeared empty. STZ-C57 mice showed the histological appearance of type 1 diabetes.

## 4. Discussion

In this study, three mice models of diabetes periodontitis were established by* P. gingivalis* oral inoculation and the metabolic and periodontal features were compared. We found no contrary impact could be observed between this periodontal infection and onset and severity of diabetes in both types 1 and 2 diabetic mice. Variations of PTPN2 and JAK/STAT pathway in gingival epithelia and different amount of serum TNF-*α* may lead to the different inflammation response to periodontal pathogen.

Currently, chemical induced and transgenic mice are two main types of diabetic models.* Db/db* mice had a long history of utilization as T2D spontaneous model. They shared the same genetic background with C57, which often used as the genetic controls to explore disease mechanisms [21]. TH mice are relatively new model for T2D characterized by glucose intolerance and hyperglycemia and show metabolic abnormalities [22]. Chemical induced diabetic mice were generally induced by streptozotocin (STZ), which destroy pancreatic *β* cells, resulting in irreversible insulin-dependent diabetes mellitus (T1D) [23].

Oral infection of periodontal pathogens has been generally used to establish periodontitis in experimental mice because of limited oral space [24].* P. gingivalis* is widely implicated as an crucial etiological agent in the pathogenesis of periodontitis [25]. The selection of bacteria with different virulence is important in making sure of the alveolar bone loss [26].* P. gingivalis* W50 is more aggressive in experimental mice for severe periodontal tissue damage because of its high virulence, compared to other strains such as* P. gingivalis* 381 and W83 [27, 28]. This research implied that* P. gingivalis* W50 could cause significant periodontal tissue destruction and alveolar bone loss in all infected mice, indicating that oral infection of W50 is an available method to establish periodontitis mice models with both types 1 and 2 diabetes.

The alveolar bone loss levels positively correlated with the severity of periodontal inflammation [29]. In our study, we could observe that all the diabetes mice exhibited alveolar bone loss and* db/db* mice exhibited more compared to other diabetes mice in both the infection and shame-infection groups. It proved that three types of diabetes mice could all exhibited the characteristic of diabetes periodontitis and* db/db* mice showed more severe periodontal infection than TH and STZ-C57 mice.

On the infection groups of all mice, no effect of periodontal infection on the fasting blood glucose was observed. However, the reverse change was found in the body weight of* db/db* mice between the infection and shame-infection groups. As* db/db* mice exhibited more severe periodontal damage, leading to the more difficulty in feeding than other mice with periodontitis, we supposed that the reverse change was elicited by the more periodontal damage, resulting in less food-intake. In all, the results revealed that periodontal infection could not affect the risk and severity of diabetes in both type 1 diabetes and type 2 diabetes mice.

Inflammation plays a central role between the pathogenesis and diabetic periodontitis. Prior research has proved that the serum TNF-*α* levels positively correlate with fasting blood glucose level, which can not only destroy pancreatic *β* cells and reduce the sensitivity of insulin but also active the signaling pathways like JAK/STAT [30, 31]. In our study, we observed that the serum TNF-*α* levels of diabetic mice were higher compared to their normal controls, and the diabetic periodontitis mice showed higher serum TNF-*α* levels than those of C57 mice in the infection group. However, the differences among three types of diabetes mice were not statistically significant. The results indicated that diabetes mice models could all represent the degree of diabetes periodontitis inflammation.

Recent reports have found PTPN2 is a regulator of inflammatory response [9]. Its crosstalk with inflammatory pathways such as JAK/STAT has been found in immune cells like astrocytes and macrophages [32]. Researches about human macrophages also demonstrated the communication between PTPN2 and JAK/STAT signaling [33]. A recent research implied that the expression of PTPN2 decreased in gingival epithelial under condition of periodontal disease [19]. Based on this research, we found the PTPN2 expression in the gingival epithelium negatively correlated with the severity of periodontal destruction and hyperglycemia. Moreover, the expression of JAK1, STAT1, and STAT3 increased in the gingival epithelium of diabetic periodontitis. JAK/STAT pathway is important in the progress of chronic human inflammatory diseases including diabetes. The main proteins in this pathway (JAK1, STAT1, and STAT3) are proven to be enhanced in periodontal inflammation [12]. Our result demonstrated that the interaction between JAK/STAT pathway and PTPN2 may contribute to the development of diabetic periodontitis. However, further comprehensive studies are needed to clarify the accurate mechanisms.

Taken together, severe periodontitis with T2D could be observed in* db/db*, while mild T2D periodontitis was found in TH mice. STZ-C57 mice exhibited characteristics of T1D. The changes of inflammation-related protein expressions in gingival epithelia might lead to the differences in the mouse models. These researches may provide the evidence of diabetic periodontitis for further study.
